# The paucity of ethical analysis in allergology

**DOI:** 10.1186/1710-1492-9-5

**Published:** 2013-02-07

**Authors:** Jason Behrmann

**Affiliations:** 1 Institute for Gender, Sexuality, and Feminist Studies, McGill University, 3487 Peel Street, 2nd floor, Montréal H3A 1W7, Canada

**Keywords:** Allergy, Asthma, Ethics, Literature review, Health policy, ELSI, Knowledge transfer

## Abstract

While a growing body of research is uncovering the aetiology and effective treatments for allergy, research that assess the broader ethical implications of this disease is lacking significantly. This article will demonstrate both the paucity of academic research concerning ethical implications in allergy and explain why ethical analysis is integral to formulating effective health strategies for allergic disease. An exhaustive literature search of publications in French and English identified less than 35 academic articles focussed on the topic of ethics and allergy; this is a miniscule number when compared to the amount of articles published on ethical issues related to other chronic illnesses, such as obesity. It is important to demonstrate to allergy specialists the need for, and utility of, further incorporating ethical analyses in allergology; the current success of Ethical, Legal, Social Implications (ELSI) research programmes in human genetics and nanotechnology will serve as notable examples. Indeed, future research and innovation in allergy will undoubtedly encounter ethical dilemmas and the allergology community should play a significant role in helping to address these issues. However, incorporating ethical analyses in allergology does not imply that the allergology community must acquire extensive knowledge in bioethics; instead, interdisciplinary research that incorporates expertise from allergology and bioethics would enable allergy specialists to advance critical knowledge development in this largely overlooked domain of study.

## Review

### Introduction

Without a doubt, the sudden development of an epidemic of a chronic disease would garner significant concern amongst the public, clinicians and health officials. It is reasonable to assume that such concern would then motivate the conduct of empirical studies to identify the underlying mechanisms of the disease in order to then evaluate possible health interventions. With such knowledge, value-based judgements and thorough debate centring on how best to prioritize and disseminate treatment options and preventive efforts would likely follow. Of additional importance would be to investigate the broader societal, ethical and legal implications (ELSI) that an emerging epidemic will inevitably raise for society. While logical, this sequence of events appears to be less-than-ideal with regards to the treatment of allergy and atopic conditions.

A seeming weakness in allergology is how the field has addressed debates concerning the implementation of treatment strategies, and the justification of value-based judgements about particular health policies. Or in other words, there is a considerable lack of analysis concerning the ethics and legitimacy of allergy research initiatives, treatments, and health policies. Consider, for example, the enactment of Sabrina’s Law in Ontario, Canada in 2006 [1]. Following the tragic death of student from an anaphylactic reaction on school premises, the provincial government mandated that all public schools must “have policies or procedures in place to address anaphylaxis in schools, which includes providing instruction to staff and guidance on the administration of medication” [1]. Though being progressive legislation that marked a significant step forward towards protecting the health of allergic students, there is little evidence that the ethical implications of this legislation was investigated prior to its implementation. Indeed, ethical assessments of food allergy policies for schools only began four years after Sabrina’s Law became formal legislation [2].

A recent editorial written by prominent researchers in allergology and published in the journal *Allergy* helps to further place the current interest towards ELSI issues in allergology into context [3]. This editorial provides an overview of several global allergy research networks and future research areas that are of foremost interest. Rather surprisingly, only investigations centring on physiological aspects of allergy were deemed of importance; no mention is made of the need for future analyses that serve to identify the broader social, legal, and ethical factors that significantly influence allergy treatment strategies and the population distribution of morbidity. The omission of the latter should not be misconstrued to imply that the broader and “less technocratic” social, legal and ethical issues of allergy have been investigated in depth and thus merit little, if any, priority.

Recent trends in health research suggest the contrary. As demonstrated by developments from the Human Genome Project throughout the 1990’s, leading geneticists understood from the beginning that rapid developments in health technologies will raise “ethical, legal, and social issues that [will] require careful attention by scientists, health care professionals, government officials, and the public” [4], p. 291. Indeed, along with greater knowledge of genetics, the ability to map human genomes raised several ethical concerns; for example, the emergence of novel means to discriminate against individuals based on their genetic makeup [4]. Cognizant of these issues, an ELSI research programme for human genetics was made an integral component of the Human Genome Project. Promoting ELSI investigations thus served to identify possible risks that may arise from genetic research, and correspondingly, develop appropriate policies to circumvent these risks in the future. In addition to emerging health technologies, similar ELSI programmes have since been developed for chronic diseases, such as the University of Pennsylvania’s newly inaugurated *Neurodegenerative Disease Ethics and Policy Program*[5]. This programme aims to “support research, education and training to identify and address the ethical and policy implications of advances in the diagnosis and treatment of neurodegenerative diseases, and work toward forming best practices for how these advances can be successfully translated into clinical practice” [5]. In light of the growing recognition of the need for ELSI studies to be integral components of health research initiatives, this article will demonstrate that concerted ethical analysis in allergology is arguably deficient, thus signifying that research in this sub-specialisation in allergy scholarship merits targeted development, and moreover, establishment of ELSI programmes within allergology.

The proposals put forth, however, will not argue that allergologists should now put aside their lab coats and focus on philosophical debates concerning “how many allergens can one fit on the head of a pin?”. Rather, the aim here is to further sensitize allergy specialists to the range of social and political factors that influence clinical practice and the implementation of research findings, and the contexts in which such research raises important ethical issues. With greater awareness of such issues, allergy specialists will be better positioned to engage in interdisciplinary research with members of the bioethics community in order to advance ethical analysis and debate in allergology. More generally, interdisciplinary research between these communities will help define the values that ought to guide decisions in health policy and public health interventions for allergy.

Scientific research and clinical experience serve to inform us of the underlying causes of disease, what the risks and benefits are concerning known treatment strategies, and whether emerging treatment modalities show promise in further reducing morbidity. It is wrong, however, to assume that scientific investigation and clinical practice in allergology – and the influence of both on health policy – exist within a purely objective, value-free space. Rather, all of these domains in health are interlinked and raise ethical questions in need of consideration [6-8]. What are the goals of allergy research, and how ought these goals define how resulting medical innovation is implemented and distributed amongst the population? Which forms of allergy morbidity are most significant and, under inevitable conditions of limited resources, which populations of allergy sufferers merit priority in targeted health interventions? What constitutes an effective treatment of atopic disorders, and what proportion of treatment strategies ought to comprise disease prevention efforts? The above are but a brief list of important ethical questions – with no simple answers – that must be subjected to ethical reflection and analysis in order to achieve a measure of consensus and recognition of legitimacy, as well as to enable political action (i.e., informed public policy). It is evident that the voice of the allergology community is essential to these discussions, and in turn, will determine society’s success in attenuating the devastating health consequences of the expanding epidemic of allergy.

But first, this article will now demonstrate the current (limited) extent of ethical analysis in allergology^a^.

## Methodology

An exhaustive literature search was conducted during the months of January to March 2012, via the Internet using the following academic search engines and online databases: GoogleScholar (http://www.googlescholar.com), PubMed (http://www.ncbi.nlm.nih.gov/pubmed), Web of Knowledge (Thompson Reuters; http://www.webofknowledge.com), CAIRN.info (http://www.cairn.info), Érudit (http://www.erudit.org), and Refdoc.fr (http://www.refdoc.fr). Manuscripts in the form of publications in academic journals written in English or French were included in the analysis; publications other than manuscripts appearing in academic journals or books (e.g., theses, institutional newsletters, conference proceedings, news articles) were excluded from the analysis.

An exhaustive search for ethics analyses concerning allergic disease and common atopic disorders was conducted using the keywords: ‘allergy’, ‘atopy’, ‘atopic’, ‘urticaria’, ‘rhinitis’, ‘dermatitis’, ‘anaphylaxis’, and ‘asthma’, which were paired with ‘bioethics’, ‘ethics’, ‘ethical’, ‘moral’, and ‘unethical’ to enable independent searches for each possible pairing of terms (e.g., ‘asthma ethics’, ‘atopy moral’, etc.). An equivalent search was repeated using the same key words in French (uticaire, rhinite, dermatite, atopie, atopique, allergie, anaphylaxie; éthique, bioéthique, morale, moraux). Manuscripts retrieved for each pair of search terms were assessed for content and inclusion in this study. Manuscripts were further excluded from analysis if they met the following criteria: 1) ethics terminology was mentioned only in passing (e.g., appear in two or fewer sentences) *and* the analytical content of the manuscript did not focus discussion on ethical issues; 2) the manuscript *only* mentioned ethics in relation to the research project having passed ethical review by an Insitutional Review Board (e.g., IRB, ethics advisory board, protocols for the ethical conduct of human subjects in research, etc.); 3) provided titles and abstracts in English or French but the text of the manuscript is of another language. The remaining manuscripts were read and further divided into two categories which determined their inclusion in the primary analysis or whether they were merely listed in a separate table in this article: 1) manuscripts of the category of academic articles are included in the primary analysis (comprising research, review, debate/discussion pieces, etc.); 2) manuscripts comprising shorter publications in the form of correspondences, letters to the editor, brief commentaries, and editorials are listed in Table 1 only and are not described in the main analysis herein. Manuscripts were deemed to be of particular interest (marked with an ‘X’) if they devote a significant discussion of ethics *in relation to* allergy (rather than limit discussion of ethical issues to a paragraph or only a short section heading within the manuscript, or if ethical issues are delegated as a distinct topic for analysis such that ethical issues are not framed particularly within the context of allergy).

**Table 1:** Manuscripts other than articles excluded from the primary analysis

**Year of publication**	**Author(s)**	**Of interest**	**Reference**
2011	Murphy, Sandel	X	[9]
	Kling	X	[10]
2010	Kling	X	[11]
	Wolf et al.		[12]
	Bleecker et al.		[13]
	Martinez, Fabbri		[14]
	Naspitz, Warner	X	[15]
2007	Payne		[16]
	Hourihane, Beirne	X	[17]
2006	Coffey, Ross		[18]
2004	Kling	X	[19]
2002	Carter		[20]
	Bisgaard et al.		[21]
	Savulescu, Spriggs		[22]
2001	Bonetta		[23]
	Bush		[24]
	Warmer		[25]
1999	Ferdman, Church		[26]
1998	Kelso		[27]
1995	Mansmann		[28]
	Eaton, Downing	X	[29]
	Reisman		[30]
1994	Smith, Burton		[31]
1993	Reisman		[32]
	Schmidt		[33]
	Frew		[34]
1987	Sly		[35]
1980	Rubenstein		[36]
***2007***	***Revuz***		[37]

Bold and italic font indicates a manuscript written in French.

In order to provide a simple comparison in the amount of ethics research available for chronic diseases other than allergy, the parameters of the literature search were repeated for obesity. However, this literature review was limited to the term ‘obesity’ (e.g., using search terms ‘obesity ethics’, ‘obesity moral’, etc.), and did not include searches employing terms for common co-morbid conditions (e.g., metabolic disorder, diabetes).

### Results and analysis: the paucity of academic articles concerning ethics and allergy

The amount of ethical reflection and research in allergology is arguably limited at best (Table 2). The results from the exhaustive literature search identified fewer than 50 academic articles on the subject of ethics and allergy, which spans 31 years of academic research (1980–2012). The majority of articles retrieved from this search (approximately 90%) have been published within the last ten years alone (2002–2012). Of these 50 articles, fewer than 35 contain a significant analysis of ethical issues in allergology (i.e., articles in which the authors provide a detailed description of ethical issues concerning allergy, rather than merely mention ethical issues within a paragraph or brief section within the manuscript; in Table 2, these articles are indentified with an ‘X’). This publication history indicates that most investigations centring on ethical issues in allergy are exceptionally recent and not representative of a sustained and long-term effort to advance knowledge in this interdisciplinary domain of study; that is to say, ethical concerns do not appear to ‘be on the radar’ of the international allergology community.

**Table 2:** Summary of results from the literature search for ethical analysis in allergy

	**Year of publication**	**Author(s)**	**Notable ethics analysis—of particular interest**	**Reference**
Articles in English	2011	Kreger et al.	X	[38]
		Behrmann	X	[39]
		Master et al.	X	[40]
	2010	Landrigan et al.		[41]
		Behrmann	X	[42]
		Behrmann	X	[2]
		Ellwood et al.	X	[43]
	2009	Engler et al.	X	[44]
		Brody et al.	X	[45]
	2008	Park, Grayson	X	[46]
		Craner	X	[47]
	2007	Scherer et al.	X	[48]
		Canonica		[49]
		Wise	X	[50]
	2006	Liss	X	[51]
		O’Lonergan, Milgrom	X	[52]
		Brody et al.	X	[53]
		Clark et al.		[54]
	2005	O’Lonergan, Milgrom	X	[55]
		Brody et al.	X	[56]
		Roberts		[57]
		Scherer et al.	X	[58]
		Resnik et al.	X	[59]
		Onder	X	[60]
	2004	Rous, Hunt	X	[61]
		Sutherland		[62]
		Dolen		[63]
		Coffey et al.	X	[64]
		Annett et al.	X	[65]
		Brown et al.	X	[66]
	2003	Brown et al.	X	[67]
		Midulla		[68]
		Brody et al.	X	[69]
	2002	Miller, Shorr	X	[70]
		Miller, Shorr	X	[71]
	2001	Payne et al.		[72]
		Holley et al.		[73]
	2000	Holt, Sly		[74]
	1996	Storrs		[75]
	1995	Harth, Thong	X	[76]
		Feingold		[77]
		Gibson et al.		[78]
	1994	Holt		[79]
	1990	Olivier	X	[80]
French articles	2009	Piette, Demoly	X	[81]
	2001	Duguet et al.		[82]
	1999	Del Volgo		[83]
	1996	Lacronique		[84]

In addition to the limited number of publications on the subject, ethical analysis in allergology has, to date, focussed discussion towards a select few domains of particular interest. Of the less than 35 articles which do devote significant ethical analysis to issues in allergology, approximately 70% of these articles target ethical issues within research contexts. Of these articles concerning research ethics, the vast majority (nearly 75%) concern research on asthma. Only 8 articles identified in this literature search conduct a significant ethical analysis on issues pertaining to public health and health policy in allergology. No books devoting chapters to ethical issues in allergy treatment or the distribution of atopic morbidities were found. The small collection of articles identified appear somewhat insular with their findings, where many articles remain separate from the others in terms of subject for ethical scrutiny. In other words, there are few links between these research publications, such that the information provided in earlier publications rarely ‘cross-fertilises’, ‘builds upon’, or cites subsequent works concerning ethics and allergy.

To date, ethical issues at the centre of analysis in allergology have an appreciable degree of variety, though many share a common theme. For example, and as mentioned above, a significant proportion of ethical scrutiny has focused on asthma research. Several publications identify particular ethical issues related to human subjects specifically [40,43,50,52,59,76], where many ethical analyses identify particular risks, benefits, and concerns of asthma investigations involving adolescent and other paediatric populations [45,46,48,53,55,56,58,64,65,69]. Additionally, allergy professionals have questioned whether it is appropriate to uphold the status quo in clinical trials for novel asthma medications. Concerns within this context primarily question if placebo controls in such trials are absolutely necessary or appropriate, since administering placebos while withholding conventional treatment regimens may expose human subjects with respiratory ailments to significant risks of harm [50,60,71]. Further publications have raised significant concerns concerning the quality and safety of clinical trials in allergology which, in addition to questions about duties to protect research subjects, aim criticism towards conflicts of interest in industry sponsored clinical trials and the publication of biased research findings [47,51,70]. Moving beyond publications related to respiratory disease, one article questions whether it remains appropriate to use human subjects for potency assessments needed for the standardisation of allergenic extracts used in immunotherapy [39].

Unlike the situation for research ethics, ethical scrutiny within clinical contexts appears limited and focuses attention towards a less diverse range of issues. One article written in French provides guidance on the appropriate use of allergy diagnostic tests amongst the population of pregnant women [81]. Certain diagnostic strategies (e.g., provocation tests) carry elevated risks of harm for pregnant women and their foetus; thus, administering such tests to this category of patients is deemed unethical and entirely contraindicated. A second article centres attention on the needs of clinicians (mainly occupational health experts), that are challenged with diagnosing occupational allergy and asthma accurately [80]. This article provides valuable guidance on how to avoid conflicts and tensions between employees with apparent occupational illnesses and companies wishing to avoid liability. A final article of clinical focus raises concern over the growing number of patients purchasing allergy and asthma treatments that fall under the heading of ‘complementary and alternative medicine’ (CAM) [44]. Primary ethical issues relate to the lack of safety and efficacy assessments for these alternative therapeutic regimens, where the article subsequently provides guidance to physicians on how best to inform patients of possibly ineffective treatments.

Similar to clinical contexts, ethical analysis in relation to health policy and public health is very limited. Two articles identify key moral issues and ethical guidelines for childhood food allergy policies at schools and child care facilities [2,61]. In terms of public health contexts, core concepts of justice are primary issues of recent scholarship. Three articles present notions of environmental justice to critique the concentration of atmospheric pollutants in impoverished neighbourhoods, which in turn impose a disproportionate burden of asthma morbidity on vulnerable populations [38,66,67]. And lastly, one article presents a public health policy assessment framework based on core principles of social justice [42]. This framework aims to provide guidance in the prioritisation of interventions to reduce environmental allergens and asthma triggers.

In addition to the publications presented in the main analysis above, this literature search also retrieved 29 very short publications comprising correspondence pieces, letters to the editor, editorials, and commentaries (Table 1). For the majority of these publications, the discussion devoted towards ethical issues is for the most part exceptionally brief and specific, and thus, it is expected that these publications are of little interest to a broad audience and do not significantly advance scholarship concerning ethics in allergology (indeed, this is typically not the goal of such publications). However, a selection of these short works are notable exceptions (Table 1; publications of interest). For example, the publications by Kling [10,11,19] provide concise overviews of core concepts in medical ethics, such as conflict of interest in research, and indicate why these issues are pertinent to investigations in allergy. These publications provide a readily tangible knowledge transfer activity useful in informing clinicians and researchers about the basics of ethical issues in allergology. A collection of editorials are also of interest in that they aim to stimulate further debate on important issues or direct greater attention towards largely overlooked topics that merit further ethical scrutiny. These topics include apparent scientific misconduct in the development of best practice guidelines for allergy treatment [29], debates over the (in)accessibility and utility of adrenaline for patients at risk for anaphylaxis [17], and the need to conduct further clinical trials that focus on paediatric populations in order to assess accurately the safety and efficacy of novel asthma treatments [15].

#### A comparison with ethics scholarship concerning obesity

Though the results from this literature review indicate ethical analysis in allergology appears quite limited, these results are not necessarily indicative of a true deficiency of knowledge or a lack of initiative in this area of study. It could be argued that ethical analysis in health science and policy (i.e., different from clinical or research ethics) is a relatively new domain of scholarship; thus, it is unsurprising that investigations concerning ethical issues pertaining to the particular disease of allergy are still in their infancy. Indeed, research in biomedical ethics only began to develop prominence in the 1960’s, and the sub-specialization of public health ethics gained notoriety at the beginning of the 1990’s [85] p. vi-viii. To address this possibility, the parameters of the literature search were replicated for the chronic disease of obesity in order to enable a simple comparison between the amount of ethics scholarship in relation to both diseases. (*For a full listing of publications cited in the following paragraph [references denoted by ‘s’], see Additional file*1*: the corresponding supplemental file for this article.)*

Obesity is a useful disease for comparison due to its similarities with allergy. Namely, both are chronic diseases that predominate in the developed world, both have a high population incidence (>25% of populations in developed countries), and both have *recently* exploded into epidemic proportions that pose a significant challenge to public health [s1-3]. From this less expansive literature search, over 60 manuscripts pertaining to obesity and ethics were identified, and accumulatively represent several hundreds of pages of published material on the subject (data not shown). Between 2007 and 2010, alone, 23 research articles were published on ethics and obesity [s4-26]. Moreover, unlike allergy where retrieved manuscripts were exclusive to academic articles, analysis of ethical issues related to obesity has been the focus of a book [s27] and the subject of several book sections [s28-35]. Comparing obesity to allergy, a reasonable conclusion derived from both literature searches is that ethical analysis concerning allergy is very limited and at an embryonic stage of academic development. Arguments that ethics in health policy and public health is too new a field of study for there to be extensive application when analysing recent epidemics of disease are not supported by these findings. Instead, the wealth of scholarship available for ethics and obesity should serve as inspiration concerning the future potential for ethics in allergology. Overall, the paucity of ethical scrutiny for allergy likely stems from other factors, such as lack of awareness, interest, or capacities to engage in interdisciplinary research that integrates ethical reflection with allergy research and clinical practice [86]. The following Discussion section will attempt to address these potential inhibitors to an applied bioethics in allergology; but first, inherent limitations of this analysis require a brief mention [86].

#### Limitations

The analysis above has notable limitations in terms of the ethics content analysis of manuscripts identified in this review. Having been assessed by the sole author of this article, decisions concerning whether a manuscript is ‘of particular interest’ is not representative of a rigorous content analysis, but rather one expert’s opinion. Thus, a degree of disaccord in these opinions is possible. With that said, the classification of manuscripts based on ethics content provided herein serves to provide a basic assessment of the extent of ethical analysis of a given publication. This ‘ethics content assessment’ aims to solely aid readers in identifying publications that may be of particular interest for future reference.

### Discussion: adding ethics to the arsenal begins with greater awareness

The provision of a broad argument supporting the need for, and utility in, applying ethical principles to aid decision-making capacities in biomedical and health contexts is not necessary for this article. For one, the vast majority of clinicians and researchers – including those specialising in allergology – are probably already well familiar with basic principles of clinical and research ethics that are now a mandatory component of most medical training curricula and that regulate practice in scientific research. The groundbreaking work by Beauchamp and Childress [87], *Principles of Biomedical Ethics* is likely familiar since it has been incorporated into numerous best practice medical guidelines. Without question, attending to principles of autonomy, beneficence, non-maleficence, and justice support good clinical practice and patient care. In terms of research, most health scientists will be familiar with the need to submit research proposals for institutional ethics review, and core principles for the protection of human subjects in research are essential elements of international laws governing human experimentation [88]. Furthermore, a recent wave of prominent ELSI scholarship has likely gained wide recognition amongst clinicians and researchers. Notable examples include ethical guidelines in health administration and the structuring of healthcare facilities [89], ethical critiques of the marketing of pharmaceuticals [90], and the identification of key sources of conflicts of interest that may compromise the quality of continuing medical education [91]. Many of the core concepts, ethical concerns, and debates advanced from such scholarship are general enough that they impinge on, or are directly pertinent to, allergy and related research. The main point made here is that though *targeted* ethical analysis in allergology is limited, it is important to note that general ethical issues of clinical practice, research ethics, health policy or scientific conduct are applicable to all fields of health, including allergology.

#### Ethical issues unique to allergology that exemplify promising areas of future research

While the above safeguards and broad ELSI scholarship are well established in legislation, in professional codes of ethics, and in medical practice guidelines, this general knowledge base is not all-encompassing. There are circumstances unique to allergology that require greater awareness, scrutiny, and debate in order to ‘fine-tune’ the decision-making capacities of clinicians and researchers. The following discussion will present three examples to exemplify ethical issues of particular significance to allergology, where these examples also serve to identify three specific areas in allergy research that merit future ethical analysis.

Consider the observation that visible minority patients in the United States, such as African Americans, are less likely to receive asthma treatment according to best practice guidelines and are less likely to receive adequate education concerning how to properly administer their asthma medication [92]. These inequalities in treatment provision do not necessarily arise because of endemic racism in medicine; instead, these inequalities might stem from patient characteristics such as socioeconomic status [9], where patients possessing a higher education level are more inclined to ask their physician necessary questions concerning their treatment [93]. Regardless, clinical allergists must be aware of the potential for inadvertent bias and thus strive to uphold principles of justice in the provision of appropriate information and asthma treatments to all patients. Future research should assess means to minimize unjust inequalities in the provision of asthma treatments and identify strategies to avoid inadvertent biases that may arise when attending to the needs of vulnerable patient populations.

Another example pertains to research, where emerging clinical trials show promise in the development of immunotherapy for food sensitivities [94]. The expected success of these trials will encourage further development of additional food allergen vaccines and novel treatment modalities. Yet, how ought future clinical trials be constructed to investigate these novel drugs and treatments, and what population(s) ought to compose the primary study group? Since food allergy and associated risks of anaphylaxis disproportionately afflicts children [95,96], ought trials focus on establishing appropriate dosing schemes for this population? While children will stand to benefit most from clinical developments from these trials, including this vulnerable population in research is typically discouraged and often encounters significant ethical challenges (e.g., informed consent with young children is often impossible) [55,97]. The allergy research community will need to debate these ethical issues. At the very least, such ethical reflection will help avoid possible challenges concerning innovation in immunotherapy and assist in securing public, academic, and political support for these much needed research endeavours.

As a final example, consider the link between technological innovation, the commercialization of novel therapies, and access to essential drugs. For many people with allergies and related atopic disorders, uninhibited access to therapeutic interventions is indispensable to achieving an appreciable quality of life. It is therefore disquieting that numerous social, legal, and political factors limit access to essential therapies. Consider recent innovations that enabled the transition to chlorofluorocarbon (CFC)-free asthma inhalers. Ozone depleting CFCs were banned in manufacturing except for the production of essential products, such as metered dose inhalers of drugs used in the treatment of chronic lung disease [98,99]. The purpose for this exception, however, was to allow time for research to uncover suitable replacements. Indeed, the discovery of novel, non-aerosol administration techniques and the propellant hydofluoroalkane (HFA) enabled a gradual phase-out of CFCs in asthma medications [100-102]. But these cumulative innovations have not been exclusively beneficial. The patenting of these novel drug administration methods has resulted in pharmaceutical companies regaining monopoly rights in the production of once common, and inexpensive, generic asthma drugs [103]. Such monopoly privileges restrict access and impose cost-barriers [104,105] to medications that many impoverished people require to live free of severe disability (elevated costs of treatment are also a major factor in patient non-compliance to therapy [106]).

Surely these turn of events were not the intended goals of the academic researchers that contributed towards developing these CFC-free drug varieties. Moreover, inadvertent restrictions in access to essential drugs runs counter to core values that the application of research knowledge should serve to benefit society while avoiding the potential for harm whenever possible. Now cognizant of these contradictions in values, researchers ought to assess whether there are more ethical strategies to transfer research knowledge into clinical application. Such strategies would likely uphold and be guided by principles of benefit maximization, harm reduction, and justice in the provision of treatment; indeed, the choices made by senior investigators and directors of research institutions can help determine the success of these laudable strategies. For one, investigators and directors of research institutes could re-evaluate conditions that define patents on innovations developed through their efforts or at their institutions. Recent policies concerning the patenting of innovations discovered at the University of British Columbia (Canada) is a notable example [107]. Known as the *Global Access Initiative*, some university polices mandate that patent rights are transferred to corporations under the condition that products commercialized from patented technology will be available to populations of the developing world. To enable such access, corporations must provide discount pricing of products destined for developing world markets. Future investigations by allergy researchers should consider devising similar policies concerning patenting and assess whether these models will uphold their core values of maximizing access to, and the benefits of, medical innovations made at their institutes.

The above examples of ethical issues in allergology demonstrate the need for specific ethical analysis in this field of health science. At a more general level, these examples also demonstrate three topics within allergology that merit future research and debate amongst allergy experts. Greater ethical scrutiny in allergology will undoubtedly uncover numerous additional issues of interest. The remaining segment of this article will now discuss tentative strategies allergy specialists and research directors could employ in order to advance knowledge in this largely overlooked area of investigation. The following recommendations aim to be readily straightforward and will focus on encouraging interdisciplinary collaborations between allergologists and experts in applied ethics. Lessons learned from the establishment of successful ELSI programmes for human genetics and nanotechnology will serve as examples for how research institutes and funding bodies can help promote ethics scholarship within allergology at a broader level.

#### Building knowledge in ethics in allergology will require interdisciplinary collaborations: Lessons from ELSI research programmes

Merging the terms ‘ethics’ and ‘allergology’ is a straightforward indication that advancing scholarship in this hybrid domain will necessitate interdisciplinary research, and thus collaborative initiatives are inevitable (e.g., the combination of neuroscience and ethics to form the field of *neuroethics*[108]). Undeniably, it would be an overly demanding claim that specialists in allergy become equally specialized in another, unrelated domain of scholarship, that is applied ethics. The need for expertise beyond a level of general awareness and interest concerning ethical issues, however, is not essential [109]. This expertise is already available through specialists in fields such as business ethics, bioethics or environmental ethics. Having raised arguments for greater awareness and interest in ethical analysis in the previous sections, this section will now discuss issues pertaining to establishing capacities to promote cross-disciplinary investigations in allergology.

With the realization of the complex aetiologies of most pathologies that challenge public health, experts agree that effective policy strategies for these diseases will require knowledge sharing between multiple disciplines in health research [110,111]. A growing call for training in health sciences to become more interdisciplinary and inclusive of academic disciplines outside of science are also voiced as strategies to improve academic training of new scientists and clinicians [112-114]. Overall, encouraging interdisciplinary research that integrates ethics and allergology would be consonant with this more general movement. Indeed, establishing greater ties between the biomedical and applied ethics communities sounds simple enough, though it does require a sustained initiative to bridge divides and build capacities that enable real collaboration.

In practice, establishing the groundwork for interdisciplinary research is not simple and many experts voice the need for greater support to foster communication and interactions across disciplines [86,110,112,115]. In particular, numerous administrative, cultural, funding allocation, and geographical factors favour research specialising in one discipline. However, there exist means to break down barriers to interdisciplinary research [110]; as noted by Robillard and colleagues [86], the establishment of dedicated ethical, legal and social implications (ELSI) programs, such as those fully integrated into genetics and nanotechnology, provide models for reforms in other domains in the biomedical sciences. These models merit further discussion here.

From the start, leaders in the field of genetics understood that the race to map the human genome would require both the promotion of capacities in science and technology as well as careful attention towards the ethical, legal, and social issues that would arise from revolutionary understanding of human genetics [4]. Such foresight motivated the National Human Genome Research Institute and the Department of Energy of the United States government to establish in 1990 a dedicated ELSI research programme for the Human Genome Project (HGP). This programme allocated over $30 million in research and education grants to fund ELSI scholarship in human genetics. Integral to the ELSI programme was the establishment of a network of leading experts in ELSI scholarship (the ELSI Working Group). This network facilitated discussion and collaboration between scientists and ELSI scholars, which in turn served to promote knowledge transfer between research bodies and facilitate the development of policy recommendations regarding the regulation of novel genetic technologies. The establishment of the ELSI programme for the HGP has since created a ‘snowball effect’. Similar ELSI programmes have been replicated in additional domains of science and technology, as exemplified by the recent establishment of an ELSI division within the National Nanotechnology Program in the United States [116]. Similar to the HGP, funding of nanotechnology research includes grants dedicated specifically for investigations concerning the broader ethical and societal concerns that may arise from nanotechnology. Such macro-level programmes that promote ELSI research in health have set precedence, one that could be replicated for allergy and related disciplines. Indeed, similar initiatives now exist, one example being a division of the Canadian Network Centres of Excellence, *AllerGen*[117]. This research network provides funding support and networking opportunities for interdisciplinary training and research in allergy, where one its three specific goals is to advance knowledge in the domain of “Public Health, Ethics, Policy and Society” [117]. The funding and network opportunities provided by AllerGen represents a step forward in the promotion of research initiatives that target the ethical, legal, and social implications within allergology.

The take-home message here is that individual clinicians and researchers do not have the sole responsibility to establish contacts and build capacities in interdisciplinary research. Rather, research institutes and departments in allergology have an equally important responsibility to establish programmes and administrative infrastructure that will favour fruitful collaborations with other domains, including applied ethics. Despite administrative, cultural, and geographical barriers to interdisciplinary research, members of the allergology community do not need to wait for broad administrative changes in their organisations and research institutes before initiating interactions with specialists in applied ethics. For one, most allergy specialists will likely have had some association and familiarity with ethicists in their place of work through evaluations of research protocols by institutional review boards, or ethics consultations within clinical contexts. The value of this established professional network should not be underestimated and should be seen as an opportunity. Merely engaging in conversations with these colleagues – outside contexts of evaluating research proposals or participating in ethical consults for particular dilemmas – would be a simple means to exchange ideas, and initiate future collaborations and shared learning opportunities.

## Conclusion

The paucity of ethical scrutiny in allergology described in this article does not aim to denote solely a weakness in this particular field of biomedical science. Rather, this analysis aims to advance the argument that fostering the development of applied ethics in allergology would enable many *strengths* and opportunities in allergy research and in the optimal design of treatment and prevention efforts. However, the fact that this literature search retrieved fewer than 35 significant articles on ethics and allergy signifies that much work remains to be done. The rapid development of bioethics scholarship over the past decade in relation to diseases like obesity, as well as the recent development of ELSI research programmes in several domains of science and technology, should serve as inspiration of the potential that lies ahead for the allergology community.

The growing awareness [6,8,85,118] that initiatives in public health, decisions in health policy, and the emergence of new technologies are laden with ethical dilemmas and political tensions signifies that decision-makers in health would benefit from enhanced skills in applied ethics. A greater awareness and sensitivity towards the broader ethical, social, and political factors in health research would also be of benefit for clinicians and investigators, including those specialising in allergy. At the very least, having the ability to identify and verbalize ethical issues in allergy would prove beneficial when members of the allergology community are called forth to provide their expert opinion concerning future policy initiatives and strategies to quell the growth of this chronic illness. In the absence of ethical reflection, one must question whether decision-makers in health are employing all the tools necessary to design optimal treatment and prevention strategies. Furthermore, a lack of academic publications that outline ethical issues that are specific to allergy raise questions as to whether policy makers are cognizant of important ethical tensions that affect clinical practice and abilities to transfer research knowledge into effective health interventions. Thus, the current paucity of academic work in ethics in allergology signifies that future imperatives in allergology should include greater collaborative efforts with members of the applied ethics community in order to advance knowledge in this largely overlooked domain of inquiry.

On a positive note, the seeming divide between ethics and allergy research and clinical practice appears to be on the cusp of change. In 2001, The European Academy of Allergy and Clinical Immunology (EAACI) called attention to major areas in clinical and research ethics that merited future intervention [19], and recommended the establishment of a European Committee on Ethics in Allergology (ECEA). And recall once again that the Canadian research network, AllerGen, allocates specific funding to support interdisciplinary training in ELSI and public health research for allergy [117]. It is unfortunate that these capacity building efforts in ethics and allergology appear to have not yet reached their full potential; this author did not find evidence (e.g., a webpage) that the ECEA has been established, and the exhaustive literature search retrieved only one article where the authors were affiliated with AllerGen [40]. Regardless, the positive position concerning ethics scholarship put forth by these prominent organizations indicates, at the very least, a nascent recognition of the need for greater knowledge in this largely overlooked area of allergology.

One can hope that the preliminary efforts towards capacity building in interdisciplinary research made by these prominent allergy organizations will provide impetus for others to follow suit. This article aims to contribute to this process by encouraging greater awareness of the untapped ethical resources that await their application towards addressing key dilemmas in allergology. Once awareness builds, it is only a matter of time before the current foundation of ethical analysis concerning allergy will grow into a diverse body of knowledge and expertise. This is not a question of if, but when; the future success in developing effective policies and public health interventions for the epidemic of allergic disease depend on it.

## Endnotes

^a^ For this article, the following definitions for ‘ethics’ and ‘ethical analysis’ apply. Ethics is a branch of moral philosophy that centres on providing guidance in decision-making contexts, as well as the evaluation of norms of conduct. Ethical reflection thus aims to define: 1) right versus wrong conduct; and, 2) distinctions that delimit ‘bad’ versus ‘good’ versus ‘best’ choices. Ethical principles and theories (e.g., uphold justice, do no harm) attempt to justify and identify appropriate norms of conduct [87] p.2. Moving beyond abstract theories of moral philosophy, the application of ethical principles as guides in real-world decision-making contexts are the mainstay of ‘applied ethics’ scholarship, common fields of applied ethics being, for example, business ethics or bioethics. ‘Ethical analysis’ comprises a diversity of research methodologies depending on the subject being investigated, ranging from empirical studies to theoretical debates. For example, ‘descriptive ethics’ investigates how members of a group currently reason and act when faced with a moral dilemma. Such investigations commonly employ scientific methods in anthropology or qualitative analysis of narratives (e.g., ‘do terminally ill patients consider euthanasia to be justified?’). Theoretical investigations typically aim to provide guidance and frameworks to guide the decision-making capacities of decision-makers (e.g., what ought one do in this particular circumstance?), or identify foreseeable conflicts that may arise from a given policy proposal. To expand, a policy proposal may centre on devising means to avoid exposure to allergens in schools by, for example, implementing a ban on peanuts. An ethical analysis of this policy, framed by the principle of ‘the fair distribution of benefits and burdens’, would raise questions of weather this ban may: 1) place an excessive burden on children that commonly consume peanuts; and thus, 2) whether school officials have a duty to minimize this burden by offering suitable meal replacements for children affected by a peanut ban (see ref: [2]).

## Abbreviations

CAM: Complementary and alternative medicine; CFC: Chlorofluorocarbon; EAACI: European Academy of Allergy and Clinical Immunology; ECEA: European Committee on Ethics in Allergology; ELSI: Ethical, legal and social implications; HFA: Hydrofluoroalkane; HGP: Human Genome Project; IRB: Institutional Review Board.

## Competing interests

The author declares no competing interests.

## Authors’ contribution

JB conceived all ideas, conducted all research, and wrote the manuscript.

## Authors’ information

JB is a post-doctoral fellow at McGill University. His research interests centre on ethical analysis of health policies and public health interventions. His doctoral thesis focused on ethics in health policy for allergy.

## Supplementary Material

Additional file 1 Supplemental references. Click here for file
