# Supplementary material for: The paucity of ethical analysis in allergology
Source: Allergy Asthma Clin Immunol. 2013 Feb 7;9(1):5. doi: 10.1186/1710-1492-9-5 (PMC3573914; doi:10.1186/1710-1492-9-5)
Supplement: Additional file 1 — Supplemental references. [file 1710-1492-9-5-S1.docx]

**Supplemental references**

1. Isolauri E, Huurre A, Salminen S, Impivaara: **The allergy epidemic extends beyond the past few decades.** *Clin Exp Allergy* 2004, **34:**1007-1010.

2. Caballero B: **The Global Epidemic of Obesity: An Overview.** *Epidemiologic Reviews* 2007, **29:**1-5.

3. Wahn U: **The Allergy Epidemic: A Look into the Future.** In *Allergy Frontiers: Epigenetics, Allergens and Risk Factors.* *Volume* 1. Edited by Pawankar R, Holgate ST, Rosenwasser LJ: Springer Japan; 2009: 3-15: *Allergy Frontiers*].

4. de Vries J: **The obesity epidemic: medical and ethical considerations.** *Science and Engineering Ethics* 2007, **13:**55-67.

5. Holm S: **Obesity interventions and ethics.** *Obesity Reviews* 2007, **8:**207-210.

6. Schulte PA, Wagner GR, Ostry A, Blanciforti LA, Cutlip RG, Krajnak KM, Luster M, Munson AE, O'Callaghan JP, Parks CG, et al: **Work, Obesity, and Occupational Safety and Health.** *Am J Public Health* 2007, **97:**428-436.

7. Stephenson RH, Banet-Weiser S: **Super-sized kids: Obesity, children, moral panic, and the media.** *The children's television community* 2007**:**277.

8. Witkowski TH: **Food Marketing and Obesity in Developing Countries: Analysis, Ethics, and Public Policy.** *Journal of Macromarketing* 2007, **27:**126-137.

9. Beghin L, Castera M, Manios Y, Gilbert CC, Kersting M, De Henauw S, Kafatos A, Gottrand F, Molnar D, Sjostrom M, et al: **Quality assurance of ethical issues and regulatory aspects relating to good clinical practices in the HELENA Cross-Sectional Study.** *Int J Obes* 2008, **32:**S12-S18.

10. Hilbert A, Ried J, Schneider D, Juttner C, Sosna M, Dabrock P, Lingenfelder M, Voit W, Rief W, Hebebrand J: **Primary Prevention of Childhood Obesity: An Interdisciplinary Analysis.** *Obesity Facts* 2008, **1:**16-25.

11. Holm Sr: **Parental Responsibility and Obesity in Children.** *Public Health Ethics* 2008, **1:**21-29.

12. Kossmann B, Ulle T, Kahl KG, Wasem J, Aidelsburger P: **Behaviour therapy for obesity treatment considering approved drug therapy.** *GMS Health Technology Assessment* 2008, **4**.

13. Lévesque L, Ozdemir V, Godard B: **Socio-ethical Analysis of Equity in Access to Nutrigenomics Interventions for Obesity Prevention: A Focus Group Study.** *OMICS: A Journal of Integrative Biology* 2008, **12:**273-278.

14. Schulte PA, Wagner GR, Downes A, Miller DB: **A Framework for the Concurrent Consideration of Occupational Hazards and Obesity.** *Annals of Occupational Hygiene* 2008, **52:**555-566.

15. Alexander SM, Baur LA, Magnusson R, Tobin B: **When does severe childhood obesity become a child protection issue.** *Medical Journal of Australia* 2009, **190:**136-139.

16. Braveman P: **A health disparities perspective on obesity research.** *Prev Chronic Dis* 2009, **6:**A91.

17. MacLean L, Edwards N, Garrard M, Sims-Jones N, Clinton K, Ashley L: **Obesity, stigma and public health planning.** *Health Promotion International* 2009, **24:**88-93.

18. Mepham B: **Use of ethical matrices in formulating policies to address the obesity crisis.** 2009.

19. Mulvaney-Day N, Womack CA: **Obesity, Identity and Community: Leveraging Social Networks for Behavior Change in Public Health.** *Public Health Ethics* 2009, **2:**250-260.

20. Stubbs JM, Achat HM: **Individual rights over public good? The future of anthropometric monitoring of school children in the fight against obesity.** *Med J Aust* 2009, **190:**140-142.

21. Throsby K: **The War on Obesity as a Moral Project: Weight Loss Drugs, Obesity Surgery and Negotiating Failure.** *Science as Culture* 2009, **18:**201 - 216.

22. Townend L: **The moralizing of obesity: A new name for an old sin?** *Critical Social Policy* 2009, **29:**171-190.

23. Varness T, Allen DB, Carrel AL, Fost N: **Childhood Obesity and Medical Neglect.** *Pediatrics* 2009, **123:**399-406.

24. Fisher CE, Devlin MJ, Appelbaum PS: **Parsing Neurobiological Dysfunctions in Obesity: Nosologic and Ethical Consequences.** *The American Journal of Bioethics* 2010, **10:**14-16.

25. Hofmann Br: **Stuck in the Middle: The Many Moral Challenges With Bariatric Surgery.** *The American Journal of Bioethics* 2010, **10:**3 - 11.

26. Meetoo D: **The imperative of human obesity: an ethical reflection.** *British journal of nursing (Mark Allen Publishing)* 2010, **19:**563.

27. Gard M, Wright J: *The obesity epidemic: science, morality, and ideology.* Psychology Press; 2005.

28. Mepham B: **The Ethical Matrix as a Tool in Policy Interventions: The Obesity Crisis.** In *Food Ethics.* Edited by Gottwald F-T, Ingensiep HW, Meinhardt M: Springer New York; 2010: 17-29

29. Sigman G: **A Child’s Right to an Environment That Prevents Obesity: Ethical Considerations.** In *A Child's Right to a Healthy Environment.* *Volume* 1. Edited by Garbarino J, Sigman G: Springer New York; 2010: 163-181: *The Loyola University Symposium on the Human Rights of Children*].

30. ten Have M, de Beaufort I, Holm S: **No Country for Fat Children? Ethical Questions Concerning Community-Based Programs to Prevent Obesity.** In *Preventing Childhood Obesity.* Wiley-Blackwell; 2010: 31-39

31. Wickins-Drazilova D, Williams G: **Ethical and public policy aspects of childhood obesity: opinions of scientists working on an intervention study.** *Obesity Reviews* 2010, **11:**620-626.

32. Korthals M: **Challenges of Genomics to Obesity and Traditional Ethics.** In *Genomics, Obesity and the Struggle over Responsibilities.* *Volume* 18. Edited by Korthals M: Springer Netherlands; 2011: 3-11.[Korthals M, Thompson PB (Series Editor): *The International Library of Environmental, Agricultural and Food Ethics*].

33. Korthals M: **Three Main Areas of Concern, Four Trends in Genomics and Existing Deficiencies in Academic Ethics.** In *Genomics, Obesity and the Struggle over Responsibilities.* *Volume* 18. Edited by Korthals M: Springer Netherlands; 2011: 59-76.[Korthals M, Thompson PB (Series Editor): *The International Library of Environmental, Agricultural and Food Ethics*].

34. Korthals M: **Prevention of Obesity and Personalized Nutrition: Public and Private Health.** In *Genomics, Obesity and the Struggle over Responsibilities.* *Volume* 18. Edited by Korthals M: Springer Netherlands; 2011: 191-205.[Korthals M, Thompson PB (Series Editor): *The International Library of Environmental, Agricultural and Food Ethics*].

35. Swierstra T, Keulartz J: **Obesity in 2020: Three Scenarios on Techno-socio-ethical Co-evolution.** In *Genomics, Obesity and the Struggle over Responsibilities.* *Volume* 18. Edited by Korthals M: Springer Netherlands; 2011: 97-112.[Korthals M, Thompson PB (Series Editor): *The International Library of Environmental, Agricultural and Food Ethics*].

36. Robillard JM, Federico CA, Tairyan K, Ivinson AJ, Illes J: **Untapped ethical resources for neurodegeneration research.** *BMC Med Ethics* 2011, **12:**9.
